# Binding Mechanism Elucidation of the Acute Respiratory Disease Causing Agent Adenovirus of Serotype 7 to Desmoglein-2

**DOI:** 10.3390/v12101075

**Published:** 2020-09-25

**Authors:** Marc-André Hograindleur, Gregory Effantin, Daphna Fenel, Caroline Mas, André Lieber, Guy Schoehn, Pascal Fender, Emilie Vassal-Stermann

**Affiliations:** 1 Univ. Grenoble Alpes, CNRS, CEA, IBS, F-38000 Grenoble, France; mahograindleur@gmail.com (M.-A.H.); gregory.effantin@ibs.fr (G.E.); daphna.fenel@ibs.fr (D.F.); caroline.mas@ibs.fr (C.M.); guy.schoehn@ibs.fr (G.S.); 2 Department of Medicine, Division of Medical Genetics, University of Washington, Seattle, WA 98195, USA; lieber00@uw.edu

**Keywords:** adenoviruses, desmoglein-2, virus-host interactions, cryo-electron microscopy, acute respiratory distress syndromes

## Abstract

The study of viruses causing acute respiratory distress syndromes (ARDS) is more essential than ever at a time when a virus can create a global pandemic in a matter of weeks. Among human adenoviruses, adenovirus of serotype 7 (HAdV7) is one of the most virulent serotypes. This virus regularly re-emerges in Asia and has just been the cause of several deaths in the United States. A critical step of the virus life cycle is the attachment of the knob domain of the fiber (HAd7K) to the cellular receptor desmoglein-2 (DSG2). Complexes between the fiber knob and two extracellular domains of DSG2 have been produced. Their characterization by biochemical and biophysical methods show that these two domains are sufficient for the interaction and that the trimeric HAd7K could accommodate up to three DSG2 receptor molecules. The cryo-electron microscopy (cryo-EM) structure of these complexes at 3.1 Å resolution confirmed the biochemical data, and allowed the identification of the critical amino acid residues for this interaction, which shows similarities with other DSG2 interacting adenoviruses, despite a low homology in the primary sequences.

## 1. Introduction

Human adenoviruses (HAdV) are non-enveloped double stranded DNA viruses. To date, 51 serotypes, and over 70 genotypes defined by bioinformatics analysis have been classified into 7 species (A–G) [1]. Although many infections are usually mild and self-resolving [2], young children, immunocompromised individuals, or individuals with some underlying diseases, might be at a higher risk for severe disease [3,4]. Transmission occurs from infected individuals to others via inhalation of aerosols (cough or sneeze), direct contact or, to a lesser extent, via fecal-oral contamination (Centers for Disease Control and Prevention, https://www.cdc.gov/adenovirus/about/transmission.html). HAdVs can be shed from the respiratory and gastrointestinal tracts for weeks or longer, even in persons who are no longer symptomatic [2]. Since adenoviruses are resistant in outdoor environments, such as high temperature and low humidity, humans can also be contaminated through contact with environmental sources (water and surfaces) [5]. Furthermore, as non-enveloped viruses, the adenoviral genome is densely packed with proteins into a particle, making adenoviruses highly resistant to many common disinfectants [6].

Most HAdV species appear to circulate globally, but predominant serotypes differ between countries and can vary temporally [7]. Note that transmission of new strains across continents may lead to the substitution of dominant HAdV serotypes [8]. HAdVs outbreaks have been documented for healthy adults in closed or crowded settings, such as military training site [9]. HAdV3, HAdV4, HAdV7, HAdV8, HAdV14, and HAdV55 are commonly linked to outbreaks, more virulent and likely to spread [10]. Among them, HAdV serotype 7 (HAdV7) seems to be the most virulent, causing severe complications and significant mortality. Most of the time, typical symptoms include mostly cough, nasal congestion, and fever, but progression to complicated pneumonia can occur in immunocompetent children and adults [11,12]. Once pneumonia develops, it can lead to acute respiratory distress syndrome (ARDS) and respiratory failure. HAdV7-associated acute respiratory infection (ARI) is characterized by prolonged high fever and strong inflammatory response with exacerbated cytokine response, causing a more severe airway inflammation [13,14]. Indeed, innate immune responses play an important role in the initial defense against adenovirus infection, but in some cases, excessive cytokine releases, such as interleukin-6 (IL-6), interleukin-8 (IL-8), or tumor necrosis factor alpha (TNF-α), have been reported to be associated with severe or fatal infections [15]. Historically, HAdV7 is one of the predominant agents causing ARD among military personnel in the United States [16]. Recent dramatic outbreaks, affecting general population, have also taken place around the world. In South America, HAdV7 was the predominant strain causing the most deaths from AdV pneumonia in the 1990s [17]. In Europe, a recombinant strain of HAdV3/7 was responsible for an ARI pediatric outbreak with fatal outcomes in 2004 [18]. Between November 2010 and June 2011, an increased incidence of HAdV7 infections was observed in Taiwan with severe clinical presentations causing the death of 32% of patients [19]. In Southern China, HAdV7d re-emerged in 2011 after an approximately 20-year absence causing ARD outbreaks in children [20]. More recently, in the United States, an unusual number of HAdV7 infections cases were reported in 2018, resulting in 11 deaths in Wanaque Center for Nursing and Rehabilitation in New Jersey [21].

Despite the significant clinical impact, there are currently no efficient approved chemotherapeutic agents to treat HAdV infections. Few data are available on adenovirus therapy, especially in immunocompetent people [22]. Although cidofovir, an antiviral with broad spectrum properties, has been shown to be effective in the treatment of HAdV infections in transplant recipients, the use of this drug remains controversial due to the high risk of nephrotoxicity [23]. However, recent studies have shown that brincidofovir, a lipid conjugate of cidofovir, can successfully treat HAdV infection in post-transplant immunocompromised patients and was generally more tolerable [24]. The only approved vaccine is a live oral enteric-coated adenovirus of serotypes 4 and 7 vaccine for U.S. military personnel only [25]. This live vaccine based on wild-type adenovirus strains, is not recommended for use in civilian population.

In such a context, a better knowledge on adenovirus entry in general and ARD causing serotypes in particular is more than ever needed. In 2011, desmoglein-2 (DSG2), a component of desmosomes carrying four extracellular domains (EC1 to EC4) has been reported to be the main receptor of HAdV of serotype 3, 7, 11, and 14 [26]. The critical entry step in the viral cycle is mediated by the adenovirus trimeric fiber and more especially by its distal globular domain called the ‘knob’. Later, a biochemical and biophysical study reported that the EC2 and EC3 domains of DSG2 were sufficient to mediate HAdV3 viral binding [27]. These data were confirmed by the structure of the HAdV3 fiber-knob (HAd3K) in complex with EC23 determined by cryo-electron microscopy (cryo-EM) [28]. This structure clearly showed that two monomers of the same HAd3K trimer are required for the binding of the two DSG2 domains. Interestingly, contrary to previous studies with the other adenovirus receptors Coxsackie and Adenovirus Receptor (CAR), Cluster of Differentiation 46 (CD46), and sialic acid [29,30,31,32], a full occupancy of HAd3K was not observed in cryo-EM whereas crystallographic data at intermediate resolution did not totally preclude this possibility [33].

In the present study, we investigate the binding mechanism of the ARD causing agent HAdV7 to the EC23 module of DSG2 using biochemical and biophysical methods. We report the atomic structure of the complex solved by cryo-EM using phase-plate providing improved high-contrast images.

## 2. Materials and Methods

### 2.1. Prokaryotic Expression Plasmid

The His-HAd7K cassette corresponding to Asp87 to Pro273 of the full-length HAdV7 fiber protein, (GenBank: AFV99273) was chemically synthetized with codon optimization for *Escherichia coli* expression by GenScript and cloned in pETDuet™-1 plasmid with a hexahistidine-tag (also called 6×His-tag) in N-terminus. Expression was performed in Rosetta (DE3) pLysS cultured at 18 °C until D0_650_ = 0.6. IPTG at 1 mM was then added and bacteria was collected 18 h after induction. Pellets were sonicated in 20 mM Tris pH 8.0–150 mM NaCl–3 mM CaCl_2_ and supernatant was recovered and clarified.

### 2.2. Mammalian Expression Plasmid

The EC2-EC3 domains of DSG2 (Swiss-Prot: Q14126.2) corresponding to Val149 to Ile386 was cloned using the restriction sites AgeI/KpnI in pHL-sec plasmid (Addgene plasmid # 99845) as described in [27]. It contains a Kozak sequence, a secretion signal sequence, and a C-terminal hexahistidine-tag (6×His-tag). This plasmid was used for mammalian cell transient transfection.

### 2.3. Transient Cell Transfection

Human embryonal kidney HEK293 F cells were maintained in FreeStyle 293 Expression medium (Gibco, Thermo Fisher Scientific) in vented Erlenmeyer flasks (Corning, Thermo Fisher Scientific) at 120 rpm, in a 37 °C, 5% CO_2_, humidified incubator. Cells were transiently transfected in flasks using Lipofectamine 2000 (Life Technologies) according to the manufacturer’s manual. The days before transfection, cells were seeded in fresh culture medium at 0.5 × 10^6^ cells/mL. When the density reached approximately 1 × 10^6^ cells/mL the cells were transfected. DNA was added to Lipofectamine 2000 in a 1:2.5 ratio, incubated at room temperature for 25 min and added to the cells. Supernatants were collected 3 days post transfection by centrifugation at 1500× *g*, 10 min at 4 °C. cOmplete, EDTA-free Protease Inhibitor Cocktail (Roche Diagnostics) was added to the cleared supernatant prior to storage at −20 °C.

### 2.4. Immobilized-Metal Affinity Chromatography (IMAC) Protein Purification

Prokaryotic or eukaryotic supernatants were pre-conditioned by the addition of sodium chloride, calcium chloride and imidazole to give a final concentration of 500 mM, 3 mM, and 10 mM, respectively. His-tagged proteins were purified by loading the supernatants onto a 1 mL His GraviTrap prepacked columns (GE Healthcare), pre-equilibrated with 20 column volumes (CV) of 500 mM sodium chloride, 3 mM calcium chloride, and 10 mM imidazole, 20 mM Tris-Cl pH8.0. Columns were then washed with 20 CV of the same buffer to remove non-specifically bound material. A second wash was performed at 10 mM (EC23) or 50 mM (HAd7K) imidazole in a similar buffer. Proteins were eluted with 10 column volumes of buffer containing 200 mM imidazole. Eluted proteins were concentrated and injected onto a Superdex 200 Increase 10/300 GL column (GE Healthcare) that was equilibrated with gel filtration buffer (150 mM NaCl, 3 mM CaCl_2_, 10 mM Tris-Cl, pH8.0). Fractions corresponding to the major peak were analyzed by SDS-PAGE and proteins were concentrated to 1 to 6 mg/mL.

### 2.5. Sedimentation Equilibrium by Analytical Ultracentrifugation Experiments (SE-AUC)

Sedimentation velocity (SV) experiments were performed on a Beckman Coulter XL-I analytical ultracentrifuge with an An50Ti rotor. Moreover, 3 mm or 12 mm path length 2-sector centerpieces cells, equipped with sapphire windows, were filled with 150 µL or 450 µL of sample, respectively, and centrifuged at 42,000 rpm at 20 °C. Sedimentation profiles were acquired using absorbance at 280 nm and interference optics. Analysis and fitting of the data was performed using the software SEDFIT and GUSSI. All values obtained with the c(s) distribution in 3 mM CaCl_2_, 10 mM Tris pH 8.0, 150 mM NaCl were converted to s20,W with SEDNTERP (version 20130813 Beta) using the measured density (1.005 g/mL) and viscosity (1.019 cp) of this buffer.

### 2.6. Negative-Stain Electron Microscopy

The standard mica-carbon preparation [34] was used with sample at 50 ng/mL. Grids were stained by sodium silico tungstate pH 7.4 and observed at 120 kV on a Tecnai T12 electron microscope. RELION 2.1 [35] was used for the two-dimensional (2D) classification. The 4 best obtained classes were calculated from around 2000 particles each.

### 2.7. Cryo-electron Microscopy

A total of 3.5 µL of sample were applied to glow discharged 1.2/1.3 Quantifoil holey carbon grids (Quantifoil Micro Tools GmbH, Großlöbichau, Germany) and they were plunged frozen in liquid ethane with a Vitrobot Mark IV (Thermo Fisher Scientific) (100% humidity, 20 °C, 6 s blot time, blot force 0). The sample was observed at the beamline CM01 of the ESRF (European Synchrotron Radiation Facility, Grenoble, France) [36] with a Titan Krios G3 (Thermo Fischer Scientific) at 300 kV equipped with an energy filter (Bioquantum LS/967, Gatan, Inc., Pleasanton, CA, USA) (slit width of 20 eV) and a Volta phase plate. Moreover, 2376 images were recorded automatically on a K2 summit direct electron detector (Gatan, Inc., USA) in super resolution mode with EPU software (Thermo Fisher Scientific). Movies were recorded for a total exposure of 3 s and 100 ms per frame resulting in 30 frame’s movies with a total dose of ~35 e−/Å2. The magnification was 215,000× (0.325 Å/pixel at the camera level). The defocus of the images varies between −0.5 and −1.0 μm. The phase plate position was changed automatically every ~110 images, which corresponds to an accumulated dose of ~50 nC on each phase plate position.

### 2.8. Image Analysis and 3D Reconstruction

The movies were first drift-corrected and binned 2 times by Fourier cropping with motioncor2 [37]. The remaining image processing was done in RELION 3.08 [38] and 3.1 [39]. CTF (Contrast Transfer Function) estimation was done with GCTF software [40]. An initial set of particles (box size of 108 pixels, sampling of 1.95 Å/pixel, data binned 6 times) was obtained by auto-picking with a Gaussian blob. After 2D classification (run1), the particles belonging to the best looking 2D class averages were used to create an ab-initio starting 3D model, which displays clear densities for two EC23 modules and a smeared one for the third EC23 module. Further 3D classification and 3D refinement resulted in a first 3D reconstruction with 3 EC23 modules (model 1). The best looking 2D class averages from run1 were then used as references for a second auto-picking, which resulted in a dataset of 874,253 particles. After extraction (box size 308 pixels, sampling of 0.65 Å/pixel), two more 2D classifications (run2 and 3) reduced the dataset to 394,450 particles. Two successive 3D classifications using model 1 as a reference (C1 symmetry, circular mask, 10 and 4 classes, respectively) allow to isolate a population of particles (230,740 in total) having strong EC23 densities. A third 3D classification (C1 symmetry, circular mask, 3 classes) was used to isolate the two populations of HAd7K-(EC23)_2_ (124,757 particles) and HAd7K-(EC23)_3_ (71,050 particles). Particles polishing, beam tilt correction, magnification anisotropy and CTF refinement per particle were then performed for both sets of particles followed by a last 3D classification (C1 symmetry, circular mask, 3 classes), which resulted in the final sets of particles (97,190, and 58,219 particles for HAd7K-(EC23)_2_ and HAd7K-(EC23)_3_ respectively). The final 3D reconstructions were calculated with C1 and C3 symmetry for HAd7K-(EC23)_2_ and HAd7K-(EC23)_3_, respectively, and resolutions of 3.3 and 3.1 Å were determined by Fourier Shell Correlation (FSC) at 0.143.

### 2.9. Model Refinement

The crystal structures of the HAd7K (PDB 3EXW [41]) and of domains EC2-EC3 of DSG2 (PDB 5ERD [42]) were rigid-body fitted inside the cryo-EM density maps in CHIMERA [43]. The atomic coordinates were then refined with ROSETTA [44] and PHENIX [45]. The refined atomic models were visually checked and adjusted (if necessary) in COOT [46]. The final models for HAd7K-(EC23)_2_ and HAd7K-(EC23)_3_ were validated with MolProbity [47].

Analysis of the interaction between the DSG2 modules and HAd7K were done with PISA [48] (‘Protein interfaces, surfaces and assemblies’ service PISA at the European Bioinformatics Institute https://www.ebi.ac.uk/pdbe/pisa/pistart.html). Any putative interaction identified with PISA for which the distance between atoms was more than 3.5 Å for hydrogen bond and 4 Å for salt bridge was discarded. The figures were prepared with CHIMERA and CHIMERAX [49]. The data collection and model statistics are summarized in Supplementary Table S1.

### 2.10. Bio-Layer Interferometry (BLI)

Measurements were performed on a BLItz instrument (ForteBio, Bohemia, New York, NY, USA). Prior to experiments, all Ni-NTA biosensors (FortéBio, USA) were hydrated in BLItz assay buffer (0.01 M HEPES pH 7.4, 0.15 M NaCl, 0.005% *v*/*v* Surfactant P20, 3 mM CaCl_2_) for at least 10 min. His-tagged HAd7K WT (wild type) or mutants (D265A and F269A) were loaded on Ni-NTA sensors in BLItz assay buffer solution for 180 s. Initial baseline were then recorded for 30 s. The association and dissociation sensorgrams with a non His-tagged DSG2 (Leinco Technologies) were monitored for 180 s at concentrations ranging from 0 nM to 1120 nM. Equilibrium dissociation constant (KD), and association (k_on_) and dissociation (k_off_) rate constants were calculated from the BLI data using the global fitting method provided in data analysis software BLItz Pro version 1.2.1.3.

## 3. Results

### 3.1. Co-Incubation of HAd7K and EC23 Leads to a Complex Formation

**Figure 1:**
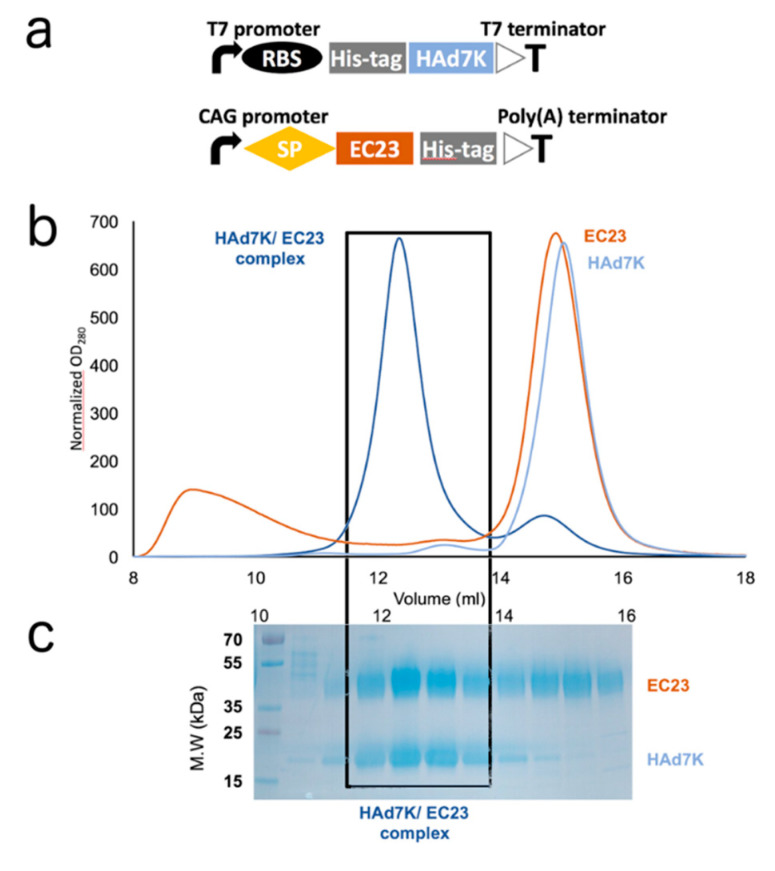
(**a**) Schematic representation of human adenoviruses, adenovirus of serotype 7 (HAd7K) pETDuet™-1 vector and EC23 pHL-sec vector constructs. RBS: ribosome binding site. SP: signal peptide; (**b**) elution profiles from size exclusion chromatography. HAd7K (light blue curve) and EC23 (red curve) were either loaded separately or co-incubated overnight (dark blue curve) before injection to the column. Complex is depicted by a box. (**c**) SDS-PAGE analysis of the eluted fractions.

Prokaryotic and eukaryotic expression systems were used for the production of the Human Adenovirus of serotype 7 knob (HAd7K) and EC23 module, respectively. By analogy with our previous work on HAd3K [27] the last repeated shaft motif before the ‘TLWT’ hinge sequence was retained for HAd7K and a histidine tag had been added at the N-terminus to facilitate purification (Figure 1a).

The HAd7K was first purified by immobilized metal affinity chromatography (IMAC) and then by size exclusion chromatography (SEC). The elution profile showed a major peak at 15.0 mL (Figure 1b) corresponding to the expected elution for the HAd7K trimer. The expression and purification of the EC23 module was performed in HEK293 mammalian cells [27,50]. The glycosylated EC23 module alone was eluted by SEC at 14.9 mL (Figure 1b). HAd7K has been described to interact with DSG2 expressed on the cell surface [26]. In order to study this interaction at the molecular level, a pure and homogeneous complex of these two partners is required. To do this, HAd7K and the glycosylated EC23 were co-incubated in a calcium-containing buffer since this cation is needed for the proper folding of the DSG2 cadherin domains [27]. Because HAd7K is trimeric and can theoretically accommodate up to three EC23 modules, the latter was added in excess. After incubation, the sample was analyzed by SEC. The elution profile shows that a peak is eluted at 12.3 mL suggesting the presence of a HAd7K/EC23 complex since each partner eluted individually around 15 mL (Figure 1b). The SDS-PAGE gel confirmed this analysis since the fractions corresponding to this peak contain both HAd7K and EC23 (Figure 1c). The excess of EC23 module in this sample is reflected by the second peak on SEC around 15 mL and is also visible on the SDS-PAGE analysis.

### 3.2. Biophysical Characterization and Visualization of the HAd7K/EC23 Complex

**Figure 2:**
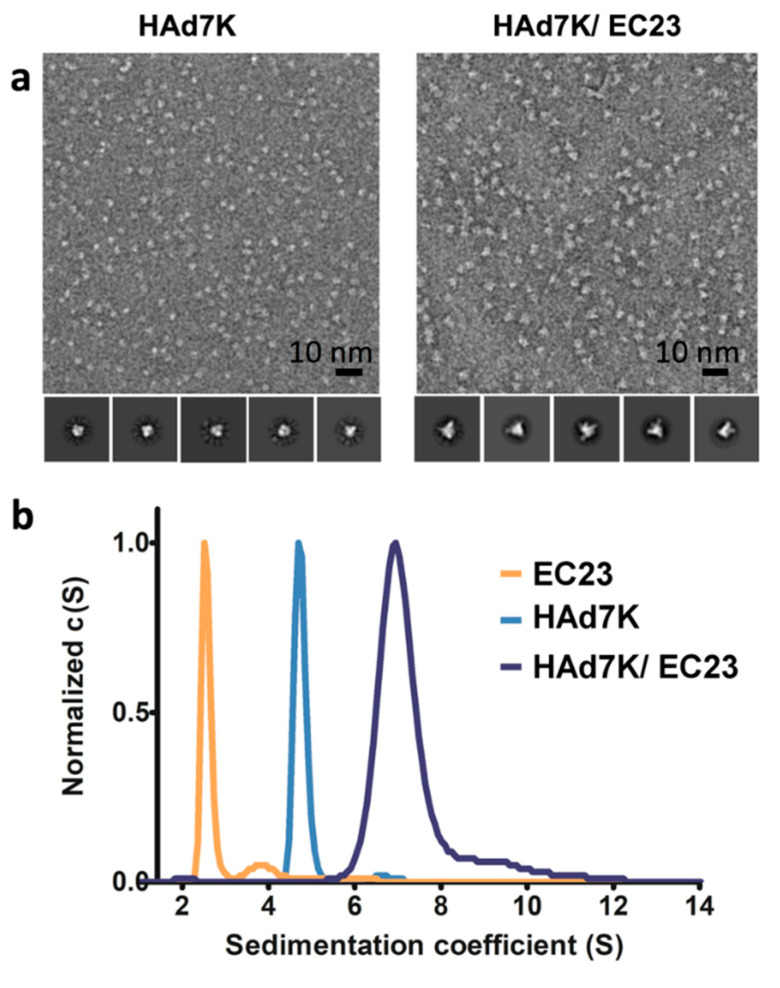
(**a**) Negative-staining electron microscopy analysis. Left: electron micrograph of HAd7K. Right: electron micrograph of HAd7K in complex with EC23. Representative class averages are shown on the galleries below each micrograph. (**b**) Analytical ultracentrifugation (AUC) analysis. Normalized sedimentation coefficient distribution profiles obtained from sedimentation velocity experiments for HAd7K (in blue), EC23 (in orange) and for HAd7K in complex with EC23 (in grey).

In order to confirm the existence of the complex and to check its homogeneity, an analysis by negative-staining electron microscopy was performed. HAd7K alone appeared homogeneous and 2D-class averages showed the expected trimer with a visible 3-fold symmetry (Figure 2a, left panel). When incubated with EC23, a homogeneous sample was also observed but with larger objects having different appearances than HAd7K alone likely reflecting the presence of EC23 (Figure 2a, right panel). The 2D-class average confirmed the presence of extra-densities due to the EC23 modules. To better characterize this complex, a biophysical method was used. The individual partners and the complex were analyzed by analytical ultracentrifugation (AUC). The sedimentation coefficient was calculated for each sample. EC23 appeared as a peak at 2.5S and the trimeric HAd7K coefficient sedimentation was assessed at 4.7S with a quite narrow peak. For the HAd7K/EC23 complex the peak was wider with the majority of the signal between 6S and 8S (average 6.9S) and a shoulder at 9.7S (Figure 2b). These observations suggested that the complex adopts several oligomeric forms.

### 3.3. Structure Determination by Cryo-EM of the HAd7K/EC23 Complex

**Figure 3:**
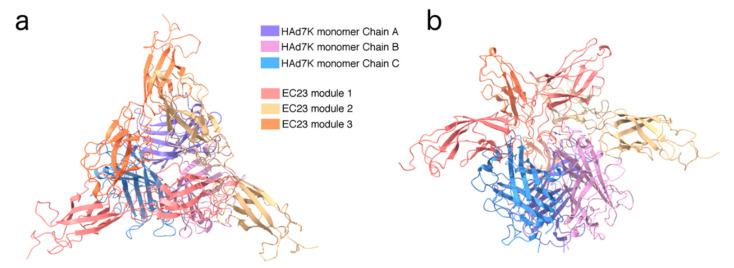
Structure of HAd7K-(EC23)_3_ viewed along the 3-fold axis (**a**) or from the side (**b**). Each monomer is colored differently according to the legend.

Images of HAd7K/EC23 were acquired by phase plate cryo-EM to enhance the contrast (Figure S1a). The 2D classification shows a well-structured complex with fine details such as the strand’s separation in HAd7K (Figure S1b). The trimeric HAd7K appears decorated with variable amount of extra densities confirming that the complex between HAd7K and EC23 might have a variable stoichiometry under the conditions used for cryo-EM. Extensive 3D classifications and refinements indeed show that the complex mainly exists in two forms, i.e., a HAd7K trimer can interact with two or three EC23 modules hereafter named HAd7K-(EC23)_2_ and HAd7K-(EC23)_3_, respectively (Figure S1c,e). There are 40% more particles in the final 3D reconstruction of HAd7K-(EC23)_2_ than in the one of HAd7K-(EC23)_3_ (Table S1). According to that statistics, it follows that the main stoichiometry of the complex by cryo-EM is two EC23 modules per HAd7K trimer. Despite all the classification steps used during image analysis, no un-decorated HAd7K or HAd7K-(EC23)_1_ class could be identified unambiguously. This does not imply that such stoichiometries do not exist in solution; but at the very least, they represent a minority of the molecules imaged by cryo-EM. HAd7K-(EC23)_2_ and HAd7K-(EC23)_3_ were solved to 3.5 and 3.1 Å resolution, respectively (Figure S1d,f), which allowed the fitting and refinement of two atomic models using the existing X-ray structures of HAd7K [41] and EC23 [42] (Table S1). The two models are very similar with the exception of the extra EC23 module in the HAd7K-(EC23)_3_ complex. The overall root mean square deviation (RMSD) between HAd7K-(EC23)_2_ and HAd7K-(EC23)_3_ (without the third EC23 module) is 0.5 Å. Each EC23 module interacts with two different monomers of the HAd7K. Furthermore, each EC domain (i.e., EC2 or EC3) interacts with one HAd7K monomer (Figure 3a,b).

### 3.4. Identification and Mutagenesis of the Key Player Amino Acids

**Figure 4:**
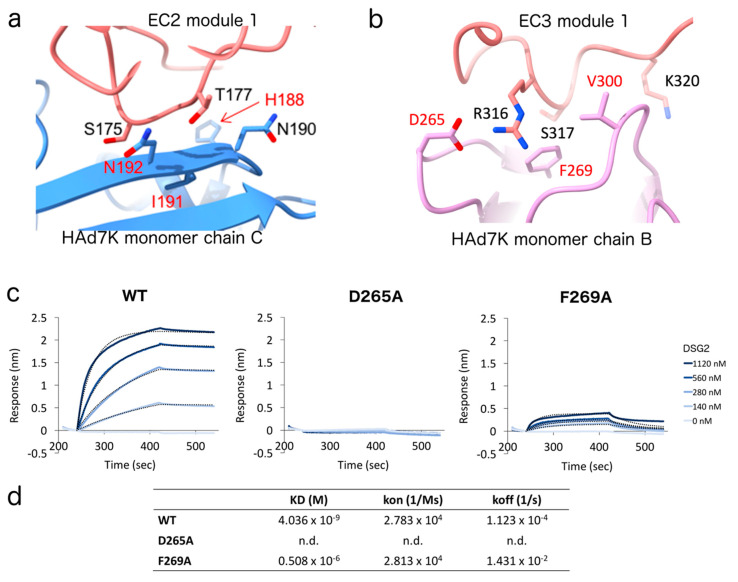
(**a**,**b**) Zoomed views of the EC2 (module 1)–HAd7K monomer chain C (**a**) and EC3 (module 1)–HAd7K monomer chain B (**b**) interactions. Side chains of key residues are represented and their names are indicated. The residues in red were found by mutagenesis to reduce by more than 80% the binding of EC23 to HAd3K. (**c**,**d**) Binding analysis of WT and mutants (D265A and F269A) HAd7K to the cellular receptor desmoglein-2 (DSG2) using bio-layer interferometry (BLI). WT or mutants His HAd7K were immobilized on a Ni-NTA biosensor, and different concentrations of DSG2 were applied in the mobile phase. The dotted lines, representing fits of the raw data (solid lines), were used to obtain equilibrium dissociation constant (KD), association (k_on_), and dissociation (k_off_) values.

A small loop in the EC2 domain which contains residues S175 and T177 interacts with a β strand of HAd7K through putative hydrogen bonds with N190 and N192 (Figure 4a). For the EC3 domain, the main interacting residue is R316 which fits into a pocket formed by two loops at the surface of one HAd7K monomer. Other EC3 residues at the interface are S317 and K320. R316 forms putative hydrogen bonds and a salt bridge with D265 while the other HAd7K residues defining the pocket are F269 and V300 (Figure 4b). Remarkably, these amino acids have previously been shown to be critical for the interaction between HAd3K, another adenovirus of the same clade, and the EC3 domain of DSG2 [28,51]. To confirm their direct contribution to DSG2 binding, amino acids HAd7K-D265 or -F269 were mutated to alanine. The mutant proteins were then purified and tested for their ability to interact with DSG2 by binding experiments. In order to quantitatively measure the binding properties of the HAd7K mutants, we performed Bio-Layer Interferometry (BLI) experiments, in which His-tagged HAd7K WT or mutants (D265A and F269A) were loaded on Ni-NTA sensors, after which DSG2 was applied as analyte (Figure 4c). Association and dissociation kinetics were then determined (Figure 4d). The data indicated that the substitution of aspartate by alanine completely abolished the binding of DSG2. To a lesser extent, F269A mutation resulted in a significant decrease in the affinity of the HAd7K for DSG2. This decrease results to an increase in k_off_ without impacting the k_on_, indicating that the mutation has a greater effect on the dissociation rate than on the association rate.

### 3.5. Comparison of HAd7K and HAd3K Binding to DSG2

**Figure 5:**
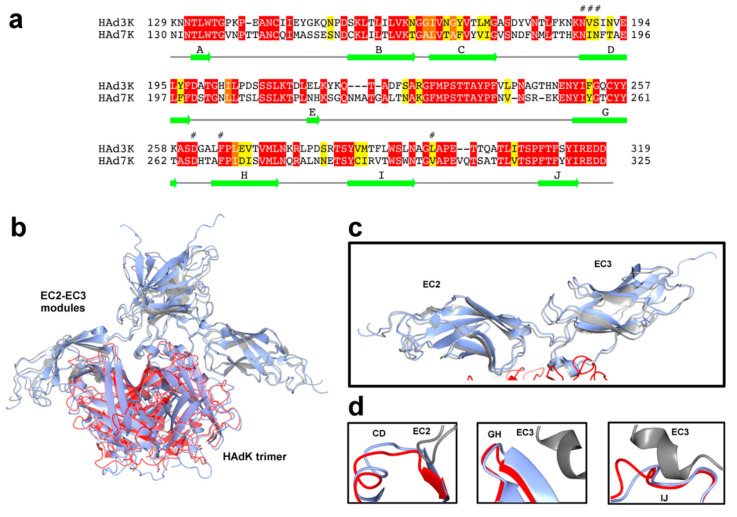
(**a**) Pairwise Sequence Alignment is used to identify regions of similarity between HAdV7 and HAdV3 fiber. For each pair of sequences the best global alignment is found using BLOSUM62 as the scoring matrix. A conservation index between 0 and 11 is calculated for each amino acid position on the alignment. Amino acids with a score above 9 are colored from red→orange→yellow corresponding to sequence conservation from high to low. (**b**) Superposition of HAd7K-(EC23)_2_ and HAd3K-(EC23)_2_: EC2-EC3 modules interacting with HAd7K (in red) are colored in grey. HAd3K-(EC23)_2_ complex is colored in blue. (**c**,**d**) Close-up views of superposed EC2-EC3 modules and superposed CD, GH, and IJ loops of HAdK, are shown individually.

The mode of interaction of EC23 with HAd7K seems conserved compared to HAd3K. Indeed, HAd3K was shown to form either a complex with one or two EC23 modules by cryo-EM [28] and even three EC23 modules by X-ray crystallography [33]. The HAd3K-(EC23)_2_ complex (PDB 6QNU) can be docked with confidence in the cryo-EM of HAd7K-(EC23)_2_ (Figure 5b). This was quite unexpected at the light of the primary sequence comparison (Figure 5a). Indeed, HAd7K and HAd3K shares only 50% identical residues regard to the fiber knob sequence. However, HAd7K can be superimposed onto HAd3K with an RMSD (Root-Mean-Square Deviation) of about 0.99 Å using 178 Ca-atoms, indicating a correct degree of structural similarity. Despite differences in sequence, surface loops and β strand identified as receptor binding site, including CD, GH, and IJ loops, have very similar conformations (Figure 5d). Major difference observed between the cryo-EM structure of the HAd3K and HAd7K lies in the IJ loop, which is extended by 2 residues in HAd7K but do not sterically interfere with DSG2. When looking at the EC23 module (Figure 5c), the RMSD between pairs of EC23 modules of HAd7K and HAd3K is 2.16 ± 0.05 Å. EC23 residues involved in the interaction with the fiber knobs of HAd7 and HAd3 are very similar.

## 4. Discussion

The expression of the HAd7K in the prokaryotic system allowed, as expected, the production of a trimeric protein as shown in the SEC experiment (Figure 1b). On the contrary, the purification of the EC23 module alone in a prokaryotic system cannot be achieved unless it is fused to a stabilizing protein such as MBP or co-expressed with the knob domain of the fiber which must act as a chaperone [27]. In this study, the EC23 module was expressed alone in mammalian cells using the (pHLsec/HEK293) secretion system [50]. It should be noted that the crystallographic structure of DSG2 revealed that each domain (EC2 and EC3) carries an N-glycosylation [42]. This suggests that the presence of these glycosylations could be required to stabilize this module. Indeed, glycosylations are visible on SDS-PAGE as shown by the smear (Figure 1c). Moreover, mass spectrometry analysis confirms this observation since, with a maximum peak at 31.974 Da for an expected protein mass of 27.823 Da, the glycosylations represent almost 15% of the receptor total mass.

Although sugars represent an important part of the EC23 module, they do not interfere negatively with the interaction with HAd7K since the co-incubation of these two partners resulted in a peak eluted earlier in SEC than the two isolated components thus reflecting the formation of a complex (Figure 1b). This sample was evaluated by negative staining electron microscopy. Despite the small size of the complex, pictures showed a homogeneous sample that differed from HAd7K alone (Figure 2a). The 2D classifications were performed showing that ‘extensions’ reflecting the presence of EC23 modules are present in the complex.

To complete this observation, an analytical ultracentrifugation (AUC) study was performed. The calculated sedimentation coefficient of HAd7K alone (4.7S) is closed to the one calculated for HAd3K in our previous study (4.4S) [27]. HAd7K in complex with EC23 resulted in two coefficients at 6.9S and 9.7S (Figure 2b), suggesting different stoichiometries. This hypothesis was confirmed by the cryo-EM analysis in which two populations corresponding to HAd7K with two or three modules were found (Figure S1c,e).

If species B adenovirus is subdivided into subspecies B:1 (HAdV3, HAdV7, HAdV16, HAdV21, and HAdV50) and B:2 (HAdV11, HAdV14, HAdV34, and HAdV35) according to their tissue tropism [52], HAdV3 and HAdV7 both belong to B:1 and are the most common strains responsible for epidemics of acute febrile respiratory disease. As the fiber is reported to be a major determinant of tissue tropism and sites of infection, the amino acid sequence of HAdV7 fiber knob was compared with the corresponding region on HAdV3 (Figure 5a). Surprisingly, while exhibiting a similar tropism, a weak identity was found between the two serotypes (about 50%). This is paradoxical, considering that a former comparison of amino acid sequences between HAdV7 and HAdV11, a urinary tract pathogen, indicated that their knob fibers share an identity of 94% [53]. Six residues of HAd3K (N186, V189, S190, D261, F265, and L296) have been shown by mutagenesis to reduce the binding of DSG2 by more than 80% [51]. The equivalent residues in HAd7K (H188, I191, N192, D265, F269, and V300) are all located at the interface with EC23. In HAd3K, contacts involving the CD loop and the D βstrand, only concerned the EC2 monomer of DSG2 and were mainly mediated by N188 and S190. In the HAd7K DSG2 complex, similar contacts with the EC2 monomer are observed, with N190 and N192. The second HAd7K/DSG2 interface involves the GH and IJ loops of HAd7K and the EC3 monomer of DSG2. The conserved aspartate residue D261, in the GH loop of HAd3K, has been shown alone to completely abrogate binding of DSG2 when mutated [28], representing thus a key anchor for the complex. Carboxyl group of HAd3K-D261 forms hydrogen bonds and salt bridge with amine group of DSG2-R316. The corresponding HAd7K Aspartate (D265) engages similar interactions with DSG2-R316. To highlight the criticality of this amino acid, a D265A substitution was introduced and the interactions between HAd7K-D265A and DSG2 were analyzed in real time. This mutation seems to modify the electrostatic properties of the side chain, preventing the interaction with DSG2. In the same way, in HAd3K, F265 contributes with Leucine (L296) to the formation of a binding pocket for DSG2. In HAd7K, phenylalanine (F269) interacts with the small hydrophobic residue Valine (V300), which is very close to Leucine in physico-chemical distance, allowing also the formation of the pocket. To study the role of Phenylalanine in the formation of the complex, a mutation was also introduced. Analysis of interactions between DSG2 and the mutated HAd7K-F269A by bio-layer interferometry, shows a drastic decrease in affinity. The data seem to be in agreement with a destabilization of the interaction. Other interfacing residues HAd3K-A297 and HAd3K-P298, forming putative hydrogen bonds with DSG2-R316, are also conserved in HAd7K (respectively A301 and P302), revealing no substantial differences in the numbers and types of contacts.

In the HAd3K-DSG2 complex, three loops of HAd3K, named CD, GH, and IJ, were found to interact with the EC2-EC3 domains of DSG2. It is known that the length of the loops could interfere with receptor binding [54]. The superimposition of the HAd7K-DSG2 structure onto HAdK3-DSG2 complex revealed that these CD and GH loops adopts similar conformations in both structures. The IJ loop is 2 aa longer in the HAd7K structure (Figure 5d) but the difference in the length of this loop does not prevent DSG2 binding to the HAdV7 fiber knob. Despite their low overall percentage of identity, HAdV3 and HAdV7 fiber knob structures reveal that their DSG2-binding regions are comparable in terms of the number and nature of contacts with DSG2. To some extent, our study indicates which amino acid of the HAdV fiber knob can tolerate modification without impacting DSG2 association. Our data also confirm that the aspartate residue (D261/D265) retained in the GH loop plays a critical role in DSG2 binding.

The HAdV7 strain analyzed here is also known as the Gomen strain, isolated as a clinical specimen from a throat washing of a military recruit with pharyngitis [55]. Since its identification, several types of HAdV7 genomes have been described showing significant intraserotypic genetic variability. Interestingly, HAdV7h, a virulent strain that has predominated in Argentina, Chile, and Uruguay, has been described as an intermediate genome 7-3 resulting from a recombination event involving DNA variants of serotypes 7 and 3. Notably, Kajon A.E. et al. showed that fiber polypeptide of HAdV7h and HAdV3 share 96.9% identity [56], indicating that most HAdV7h fiber has been replaced by an HAdV3-like fiber.

## 5. Conclusions

In this work, we reveal a new cryo-EM structure of the HAdV7 fiber knob in complex with its cellular receptor, DSG2. Using an in vitro approach, we identified point mutants of HAd7K having a strongly reduced ability to interact with DSG2, thus, elucidating the main structural requirements for fibre-DSG2 interactions. Interestingly, we have highlighted that fiber knobs from HAdV7 and HAdV3, two serotypes from subgroup B, while displaying clear differences in their primary sequence, this had not major impact on the DSG2 binding properties. Our findings contribute to a better understanding of the virus attachment, providing possible targets for the development of novel antiviral drugs.

## Data Availability

The atomic coordinates and the cryo-EM map have been deposited in the PDB and EMDB. Coordinates for the two complexes have the PDB accession codes 7AGG (HAd7K with two EC2-EC3 modules) and 7AGF (HAd7K with three EC2-EC3 modules). The two cryo-EM maps have the EMDB accession codes EMD-11779 and EMD-11778, respectively.

## Supplementary Materials

The following are available online at https://www.mdpi.com/1999-4915/12/10/1075/s1, Figure S1: Cryo-EM micrograph, reconstruction and FSC calculation, Figure S2: Zoomed views of the 3D reconstructions, Table S1: Summary of data collection and atomic model statistics.

## Abbreviations

ARDS	Acute Respiratory Distress Syndrome
ARI	Acute Respiratory Infection
BLI	Bio-Layer Interferometry
CAR	Coxsackie and Adenovirus Receptor
CD46	Cluster of Differentiation 46
Cryo-EM	Cryo-Electron Microscopy
DSG2	Desmoglein-2
HAdV	Human Adenovirus
HAdK	Human Adenovirus Knob
EC23	Extra Cellular domains 2 and 3 (from DSG2)
IMAC	Immobilized-Metal Affinity Chromatography
RMSD	Root Mean Square Deviation
