# Supplementary material for: Binding Mechanism Elucidation of the Acute Respiratory Disease Causing Agent Adenovirus of Serotype 7 to Desmoglein-2
Source: Viruses. 2020 Sep 25;12(10):1075. doi: 10.3390/v12101075 (PMC7599583; doi:10.3390/v12101075)
Supplement: Supplementary file 1 [file viruses-12-01075-s001.pdf]

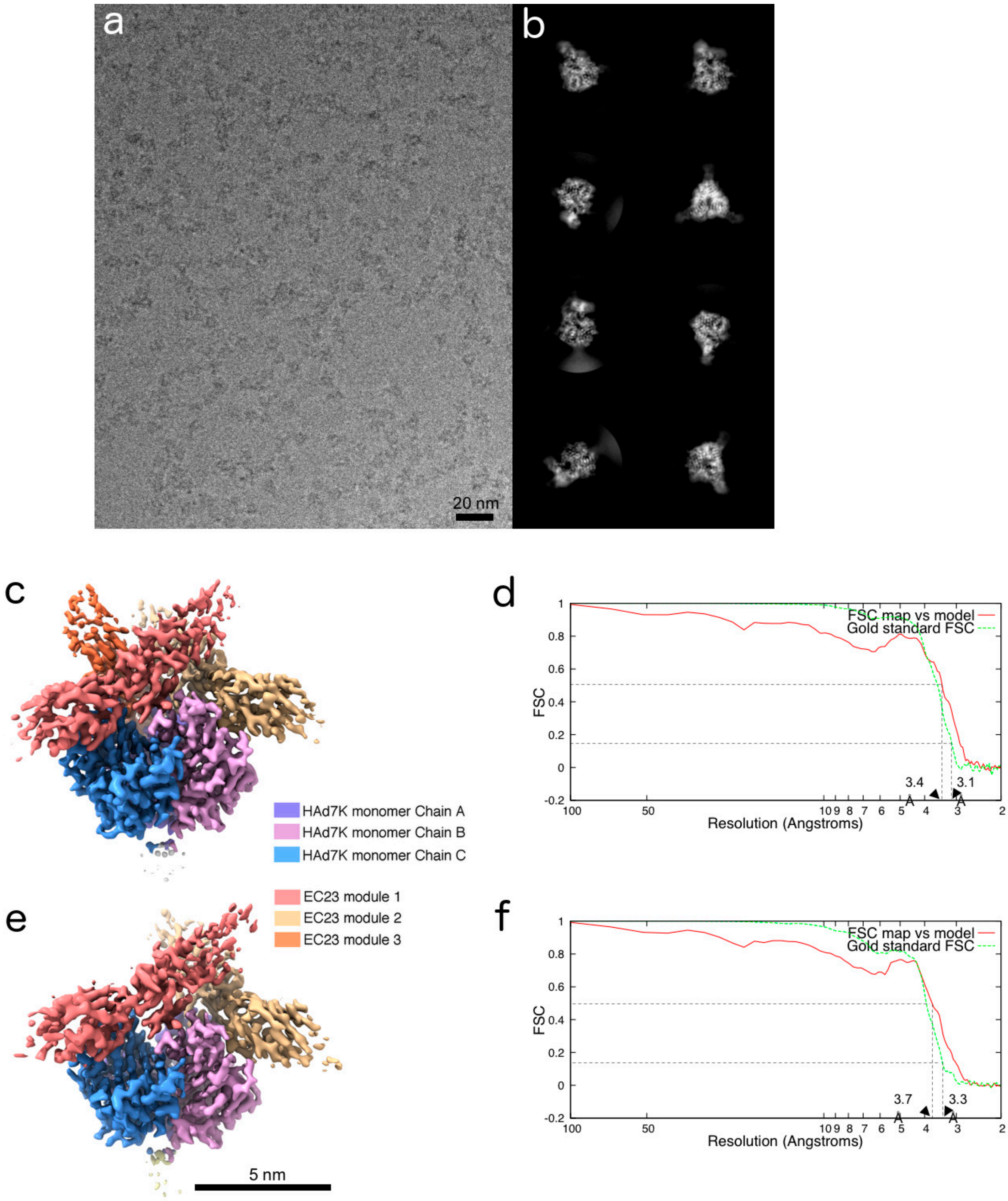

Figure S1: Cryo-EM micrograph, reconstruction and FSC calculation **(a)**: Cryo-EM micrograph of HAd7K/EC23. **(b)**: 2D class averages of HAd7K/EC23. **(c, e)**: Isosurface representations of the cryo-EM 3D reconstructions of HAd7K-$(EC23)_3$ **(c)** and HAd7K-$(EC23)_2$ **(e)** colored by monomer. **(d, f)**: Fourier Shell Correlation (FSC) curves for HAd7K-$(EC23)_3$ **(d)** and HAd7K-$(EC23)_2$ **(f).**

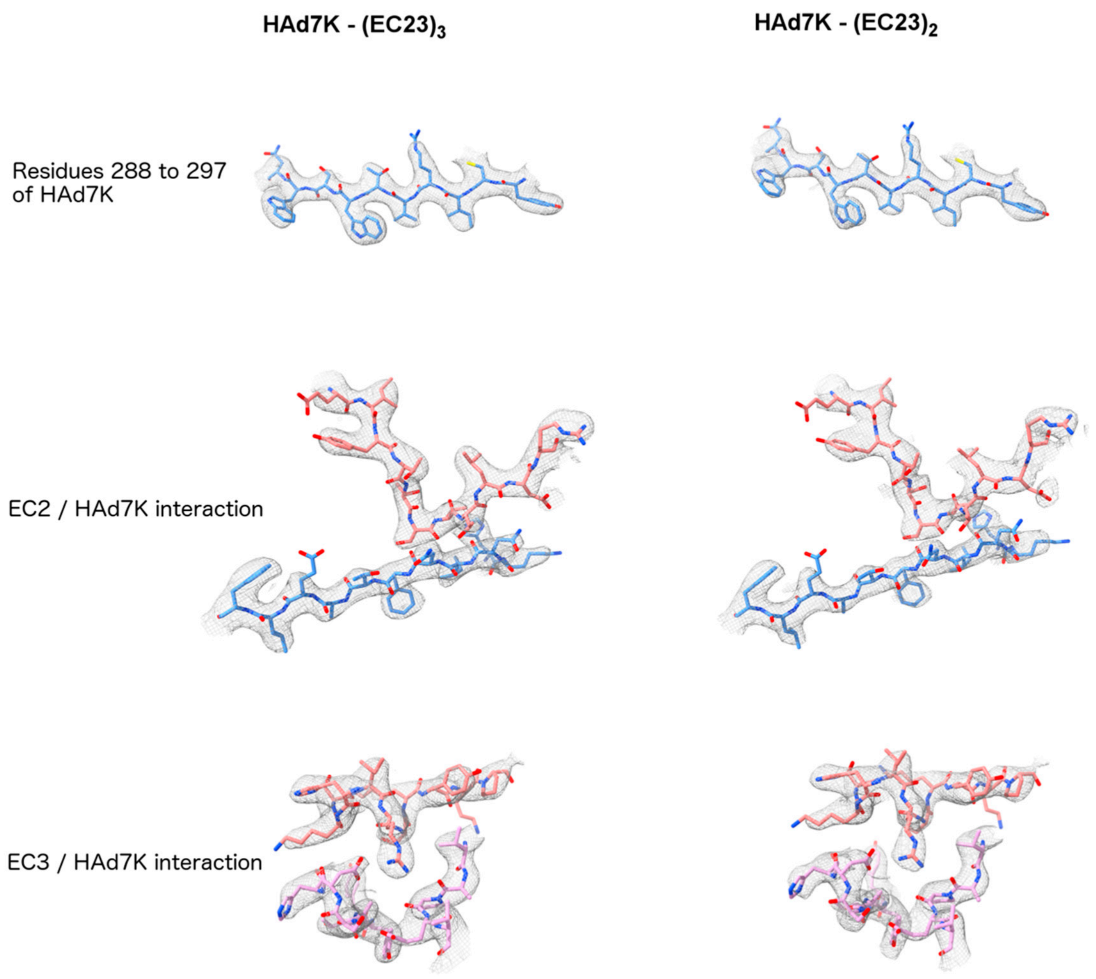

Figure S2: Zoomed views of the 3D reconstructions. Views on 3 different areas of HAd7K-$(EC23)_3$ (left column) and HAd7K-$(EC23)_2$ (right column). The cryo-EM density is represented in light grey. First row focuses on one β strand in HAd7K, second row on the interaction between EC2 (salmon) and HAd7K (blue) and third row on the interaction between EC3 (salmon) and HAd7K (pink).

Table S1. Summary of data collection and atomic model statistics

**Data collection**		
**Microscope**	**Krios G3 (ThermoFischer Scientific)**	
Voltage (kV)	300	
Magnification	215,000x	
Unbinned pixel size	0.325 Å/pixel	
Camera	K2 Summit (Gatan Inc)	
Exposure time	3s	
Number of frames	30	
Total dose (e^-/Å^2)	35	
**Image processing**		
	Had7k-$(EC23)_2$	Had7k-$(EC23)_3$
EMDB		
Symmetry	C1	C3
Final number of Particles	97,190	58,219
Map resolution in Å (FSC 0.143)	3.3	3.1
**Model statistics**		
PDB		
Model resolution in Å (FSC 0.5)	3.7	3.4

Ramachandran favored (%)	95.8	96.0
Ramachandran outliers (%)	0.2	0.8
Rotamer outliers (%)	0	0
C-beta deviations	0	0
Rms on bond lengths	0.0072	0.0063
Rms on bond angles	0.78	0.88
Clashscore	6.95	5.29
Molprobity score	1.67	1.56
